# Total body irradiation delivered using a dedicated Co‐60 TBI unit: Evaluation of dosimetric uniformity and dose verification

**DOI:** 10.1002/acm2.14188

**Published:** 2023-11-01

**Authors:** Jay W. Burmeister, Todd Bossenberger, Adrian Nalichowski, Ahmad Hammoud, Geoff Baran, Michael M. Dominello

**Affiliations:** ^1^ Department of Oncology Wayne State University School of Medicine Detroit Michigan USA; ^2^ Gershenson Radiation Oncology Center Barbara Ann Karmanos Cancer Institute Detroit Michigan USA

**Keywords:** in‐vivo dosimetry, TBI, TBI dosimetry, total body irradiation

## Abstract

This work presents the dosimetric characteristics of Total Body Irradiation (TBI) delivered using a dedicated Co‐60 TBI unit. We demonstrate the ability to deliver a uniform dose to the entire patient without the need for a beam spoiler or patient‐specific compensation. Full dose distributions are calculated using an in‐house Monte Carlo treatment planning system, and cumulative dose distributions are created by deforming the dose distributions within two different patient orientations. Sample dose distributions and profiles are provided to illustrate the plan characteristics, and dose and DVH statistics are provided for a heterogeneous cohort of patients. The patient cohort includes adult and pediatric patients with a range of 132–198 cm in length and 16.5–37.5 cm in anterior‐posterior thickness. With the exception of the lungs, a uniform dose of 12 Gy is delivered to the patient with nearly the entire volume receiving a dose within 10% of the prescription dose. Mean lung doses (MLDs) are maintained below the estimated threshold for radiation pneumonitis, with MLDs ranging from 7.3 to 9.3 Gy (estimated equivalent dose in 2 Gy fractions (EQD_2_) of 6.2‐8.5 Gy). Dose uniformity is demonstrated across five anatomical locations within the patient for which mean doses are all within 3.1% of the prescription dose. In‐vivo dosimetry demonstrates excellent agreement between measured and calculated doses, with 78% of measurements within ±5% of the calculated dose and 99% within ±10%. These results demonstrate a state‐of‐the‐art TBI planning and delivery system using a dedicated TBI unit and hybrid in‐house and commercial planning techniques which provide comprehensive dosimetric data for TBI treatment plans that are accurately verified using in‐vivo dosimetry.

## 1 INTRODUCTION

Total body irradiation (TBI) is an important component of the conditioning regimen for hematopoietic stem cell transplantation. TBI delivery equipment and techniques vary substantially as most institutions do not have a dedicated treatment unit for the delivery of TBI. In 2016, the GammaBeam 500 (Best Theratronics, Inc., Kanata, ON, Canada) became the first FDA approved dedicated TBI unit. The design and characteristics of this unit, along with details of its commissioning and operation, have been described previously.^1^ While dosimetric calculations and measurements on and within an anthropomorphic phantom have been presented,^1^ those from patient treatments have not yet been published. Here we evaluate the dosimetric characteristics of TBI plans for the initial 22 patients treated with a high dose, multi‐fraction TBI regimen on this dedicated TBI unit.

AAPM Report #17 “The Physical Aspects of Total and Half Body Photon Irradiation”^2^ and the American College of Radiology/American Society for Radiation Oncology Practice Guidelines for TBI^3^ provide guidelines for the planning and delivery of TBI and recommend a dose uniformity of ±10% about the prescribed dose. More recent guidelines provided by the International Lymphoma Radiation Oncology Group (ILROG) state that “differences in separation along the patient's length can result in dose heterogeneity that can exceed 10%−20%” and recommend the construction of a patient‐specific compensator for each patient with a desired dose uniformity goal of ±5%.^4^ Similarly, the Netherlands Commission on Radiation Dosimetry (NCS) code of practice for TBI suggests a tolerance between 5% and 10% as a warning level with a 15% tolerance for intervention,^5^ based on previously published reports.^6^, ^7^ Both end‐to‐end testing and in‐vivo dosimetric measurements have traditionally been recommended as methods to verify delivered dose distributions.^8^ Prior studies have discussed the use of diode dosimetry for TBI and demonstrated the accuracy of this technique for in‐vivo dosimetric verification and determination of midplane doses.^9^, ^10^, ^11^, ^12^, ^13^, ^14^

Since radiation pneumonitis (RP) is the most important potential treatment complication, the vast majority of facilities have a mechanism for reduction of lung dose.^15^, ^16^, ^17^, ^18^ For high dose regimens (≥12 Gy), lung shielding is commonly employed to reduce the mean lung dose to 8–10 Gy and has been demonstrated to reduce the likelihood of radiation pneumonitis.^4^, ^19^, ^20^, ^21^, ^22^, ^23^, ^24^, ^25^ While mean lung dose (MLD) has been shown to correlate with RP,^26^, ^27^ other dose‐volume parameters have also been shown to be predictive of RP.^28^ Reduction of the MLD has been shown to also be correlated with improved pulmonary function^29^ and improved survival,^30^ and MLD > 8 Gy has been shown to be associated with inferior relapse‐free survival in pediatric patients.^31^

Since most institutions do not have a treatment unit dedicated specifically for TBI, conventional linac delivery systems are typically modified to facilitate the delivery of a uniform dose of radiation to the entire patient. Recent publications have highlighted the resulting substantial heterogeneity across TBI techniques in clinical practice.^15^, ^16^, ^17^, ^18^ It is unclear how these differences in technique may affect outcome, making it difficult to interpret the results from of clinical studies. The acquisition of sufficient treatment planning and delivery data will help facilitate a better understanding of the effects of planning and delivery techniques on clinical outcome. As an example, we present a thorough evaluation of our treatment planning and delivery data for this dedicated TBI unit. While previous publications have provided estimates of total body and mean lung dose as well as in‐vivo dosimetry results for TBI, Monte Carlo calculations of dose distribution, dose‐volume, and point dose data, along with associated verification measurements, have not been previously reported. We demonstrate here our technique for treatment planning, treatment delivery, and reduction of lung dose, including treatment plan statistics and example distributions. We also demonstrate the ability to achieve ±5% dose uniformity for the majority of evaluated locations without the need for construction of a patient‐specific compensator, and provide dose and dose‐volume data for a cohort of patients treated with state‐of‐the‐art TBI using a commercial dedicated TBI unit.

## 2 METHODS AND MATERIALS

Patients treated with a high‐dose TBI regimen at our institution typically receive 12 Gy in six fractions over three consecutive treatment days. Within the cohort presented here, 18/22 patients received 12 Gy in six fractions over three days and the remaining four patients received 12 Gy in eight fractions over four days. Patients receive two CT scans from head to mid‐femur, one in the supine and one in the prone position. Scans are imported into a commercial treatment planning system (Eclipse v16.1, Varian Medical Systems, Palo Alto, CA) for field definition and design of lung blocks. Lung blocks are contoured in a manner similar to that described in the ILROG guidelines,^4^ however, since our patients are treated in the prone and supine positions, we create a pair of lung blocks for each position from the digitally reconstructed radiographs obtained from the supine and prone CT scans. The planning geometry and image data are then sent to an in‐house developed MATLAB (Mathworks, Natick, MA) program which uses DOSXYZnrc to generate a Monte Carlo dose distribution based on patient‐specific anatomy for the prone and supine positions both with and without lung‐blocking.^32^ The accuracy of an earlier version of this system has been demonstrated previously^33^ and similar dosimetric validation has been performed for the current system. The four calculated dose distributions (supine and prone, both with and without lung blocks) are then exported back to Eclipse for creation of a cumulative plan which includes each of these distributions weighted according to the number of treatment fractions that technique will be used, and for evaluation of this cumulative plan. Each MC dose distribution is normalized to a manual TAR calculation at the prescription point, and the accuracy of the manual calculations has been validated in regular and anthropomorphic phantoms using multiple dosimetric techniques, including external validation through the Imaging and Radiation Oncology Core. Since the cumulative plan is the sum of dose distributions calculated within two different patient orientations (supine and prone), deformable image registration using a modified B‐spline algorithm (Velocity v3.1, Varian Medical Systems, Palo Alto, CA) is used to map the prone scan and its associated dose information onto the supine scan. Characteristics of lung block shapes drawn in Eclipse are extracted from the DICOM plan information and used to create Cerrobend lung blocks with patient‐specific geometry and divergence.

For the first five patients treated with this treatment unit and planning system, a feet‐first CT scan was also acquired for both supine and prone setups and the two scans in each orientation were stitched together to allow the calculation of the dose distribution throughout the entire body. Following analysis of these dose distributions, along with manual calculations and in‐vivo dosimetry results, this process was discontinued in favor of the single scan to mid‐femur for each orientation. This decision was based on both the accuracy of calculations and associated dosimetric measurements, and the relative complexity of the full body scan technique. Manual calculation and dosimetric verification were performed for calculation points in the legs for the remaining patients in this study.

Dose volume histograms (DVHs) are evaluated for the entire calculated patient volume and for the lungs and heart. The lung block thickness was chosen to be 2.5 cm and blocks were used for either 4/6 or 5/6 treatment fractions based on physician preference, usually with the intent to keep the MLD below 9 Gy.^1^ The treatment delivery time is calculated based on dose to mid‐depth at the calculation point which is 20 cm superior to the umbilicus along the superior‐inferior axis of the patient. This point is chosen as it is typically close to the center of mass of the patient and the thickest part of the patient in the anterior‐posterior direction. In addition, the patient surface is more regular in shape than at the umbilicus, and thus easier to measure the source to surface distance. In addition to this calculation point, four other calculation points are evaluated, including the mid‐depth doses at the suprasternal notch, head, knees, and ankles. In addition, doses are calculated at a depth equivalent to the inherent buildup in the diodes used for in‐vivo dosimetry for both the supine and prone orientations at all five of these locations. These are then compared to diode measurements made using a VeriDose diode (Fluke Biomedical, Cleveland, OH) with inherent 1.36 g/cm^2^ buildup. Effects of SSD (dose rate) dependence have been measured to be less than 1% over the range of measurements in this study and are therefore neglected. Temperature dependence effects on the diode reading are estimated to be approximately 1.5%. Published data suggests that the equilibrium temperature for this diode is approximately 28°C and that the diode reaches 90% of this temperature within 3−5 min.^34^ Based on this, we have assumed an estimated average temperature increase in diode temperature of 5 degrees in comparison to calibration measurements made at room temperature. We measured an increase in signal of approximately 0.3% per degree for this diode in this treatment beam, suggesting an increase in response of approximately 1.5% for patient measurements. This increase has been incorporated into the measurements presented here. Angular dependence of the diode was found to result in a 1−3% increase in reading as the angle was changed from 0 to 45 degrees from perpendicular to the beam. However, every effort was made during diode placement to keep the flat side of the diode in contact with the skin and perpendicular to the beam. As such, effects of angular dependence have been assumed to be negligible for these measurements.

All patients in this cohort were treated on the Best Theratronics GB500 with the maximum head height of 247 cm which gives an SSD of 229 cm to the top surface of the treatment couch. All were treated using 2.5 cm thick custom Cerrobend lung blocks designed to match the shape and divergence of the lungs and placed on an acrylic lung block tray which mounts to the couch rails and holds the blocks 35 cm above the couch surface. One patient had a separation larger than 35 cm and required the construction of a custom acrylic holder which raised the tray to 40 cm. Lung block placement was verified using the a‐Si panel imager contained within the treatment couch. The GB500 has a flattening filter which provides a uniform dose distribution at the level of the couch over a 225 × 78 cm^2^ field size. Beam profiles within a geometric phantom have been presented previously.^1^ Patient thickness at various locations and SSD to the setup location was verified for each patient prior to each treatment fraction.

## 3 RESULTS

**FIGURE 1:**
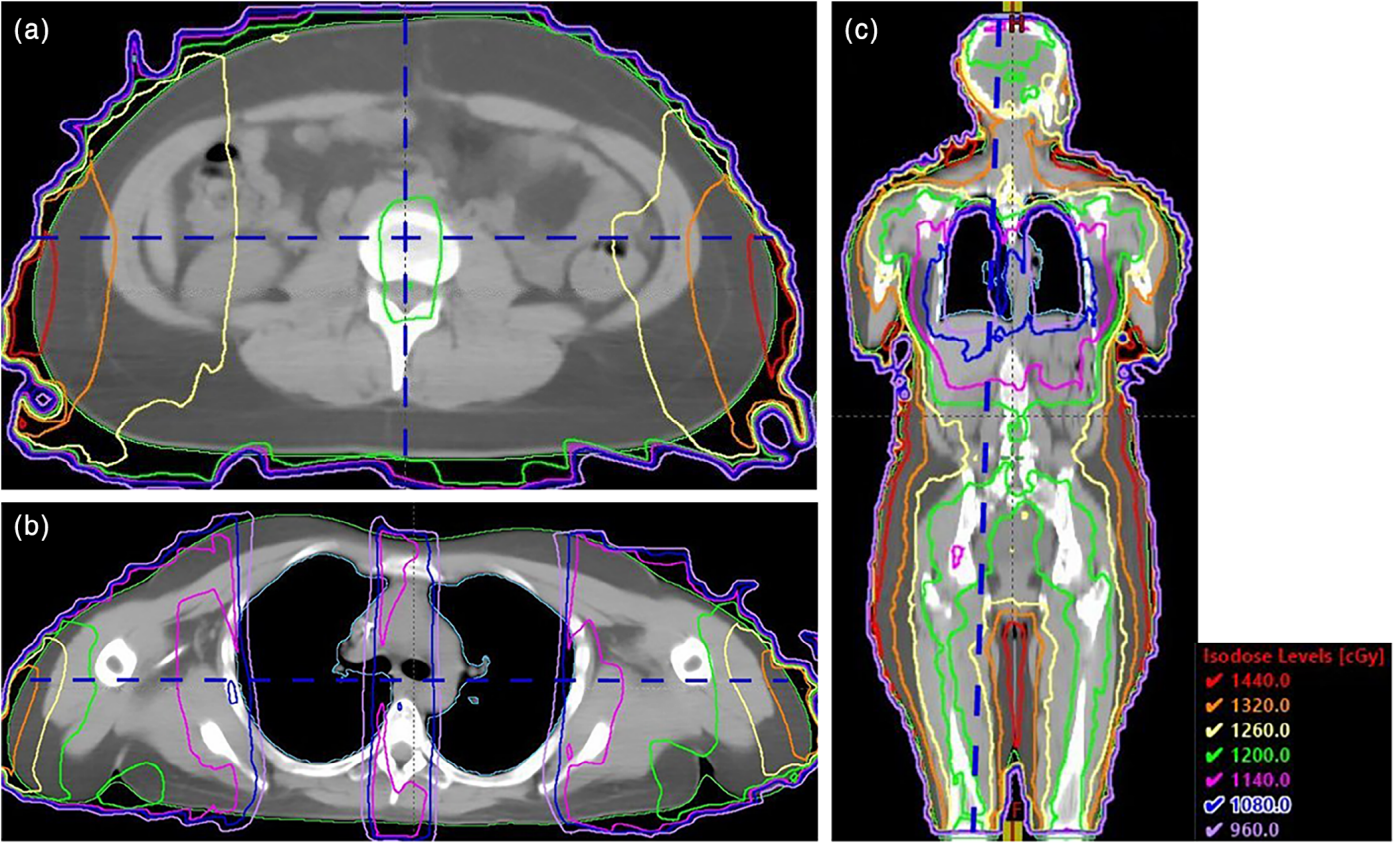
Dose distributions for a sample patient in the axial plane through the prescription location (a), in the axial plane through the lungs (b), and in the coronal plane at mid‐depth in the patient (c). The blue dashed line in Figure 1(a) represents the prescription depth.

**FIGURE 2:**
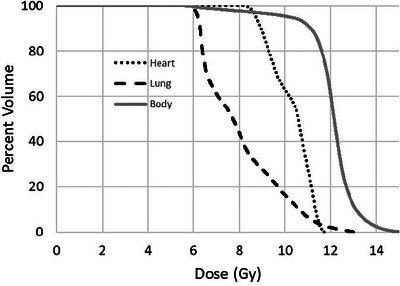
Dose volume histograms for a sample patient.

The patient cohort included both pediatric and adult patients and varied substantially in length and thickness. In the supine orientation, mean patient length was 179.6 cm (range 132–198 cm) and mean thickness at the calculation point was 24.1 cm (range 16.5‐37.5 cm). Sample dose distributions within an anthropomorphic phantom with and without lung blocks have been presented previously.^1^ Sample dose distributions for one patient in this cohort of average size and length are provided here. Figure 1(a)‐(c) illustrate typical dose distributions in the axial plane through the prescription location (a), in the axial plane through the lungs (b), and in the coronal plane at mid‐depth (c). The blue dashed lines in Figure 1(a) represent the prescription location. Corresponding DVHs for body, lung, and heart for this patient are provided in Figure 2. The MLD for this patient was 8.0 Gy, mean heart dose was 10.3 Gy, and mean dose to the remainder of the body was 12.0 Gy.

**FIGURE 3:**
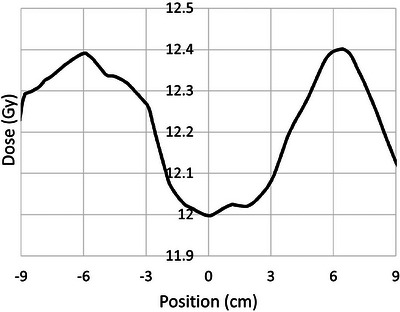
Anterior‐posterior dose profile across the vertical dashed line in the axial dose distribution shown in Figure 1(a).

**FIGURE 4:**
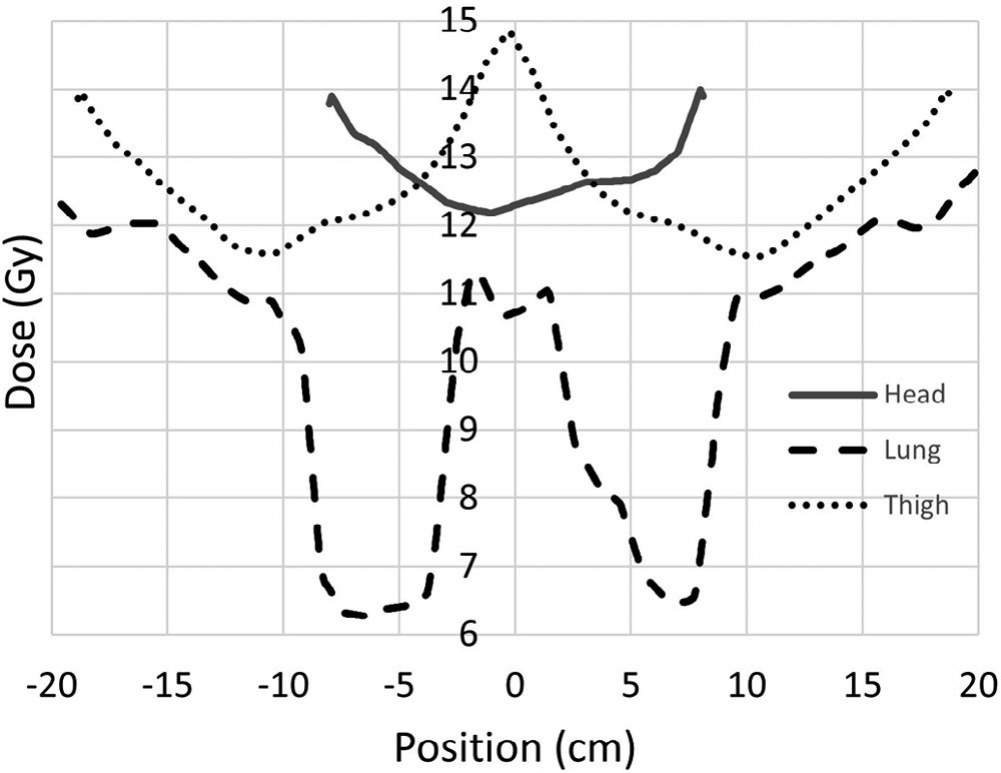
Right‐left dose profiles in the axial plane across the center of the head, lungs, and thighs. The lung profile represents dose along the horizontal dashed line shown in Figure 1(b) .

**FIGURE 5:**
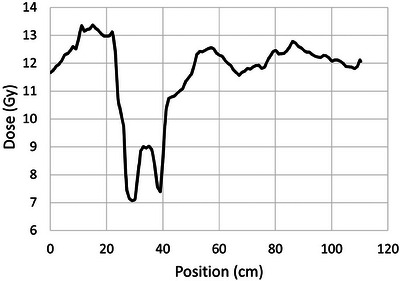
Superior‐inferior dose profile along the blue dashed line through the patient dose distribution in Figure 1(c).

The DVH in Figure 2 shows approximately 85% of these patients receiving a dose within ±10% of the prescription of 12 Gy, and 95% within ±20%. Deformation of the prone dose distribution to the supine scan does not result in a perfect match and a substantial portion of the volumes showing high or low dose are an artifact of the resulting areas of mismatch. This occurs primarily in the extremities and in areas with sharp contours. Nearly the entire central region of the body is within ±10% of the prescription dose. As an example, Figure 3 shows an anterior‐posterior profile of dose along the vertical dashed line through the axial dose distribution shown in Figure 1(a), illustrating uniform dose well within 5% of the prescription dose across the entire profile. Figure 4 shows right‐left profiles across the head, the center of the lung, and the mid‐thigh. Figure 5 shows a superior‐inferior profile along the blue dashed line through the coronal dose distribution shown in Figure 1(c), illustrating relatively uniform dose within approximately 5% of the prescription dose over the length of the body with the exception of the blocked lung region and the high surface gradient region in the neck. The profile location is slightly off the central axis and angled to allow visualization of the dose distribution through the head, lungs, abdomen, and thigh.

The mean MLD for the entire patient cohort was 8.3 Gy (range 7.3‐9.3 Gy). An α/β value of 3 can be estimated from existing literature,^35^, ^36^, ^37^ and using this value the mean MLD yields an equivalent dose in 2 Gy fractions (EQD_2_) of 7.3 Gy and the maximum MLD yields an EQD_2_ of 8.5 Gy. The EQD_2_ for the example patient shown here was 6.9 Gy. The dose rate at the calculation point for this patient cohort ranged from 7.5 to 15.5 cGy/min, with an estimated dose rate in the center of the lung of approximately 5−11 cGy/min.

Mid‐depth doses calculated at each point are presented as a ratio to the prescription dose in Table 1. The prescription point is not included here since the doses were normalized to that point for each patient. Mean doses at all calculation points are within one standard deviation but there appears to be a trend toward higher calculated dose at points other than the prescription point. Doses at all locations are well within our prescription tolerance of ±10%.

**TABLE 1:** Ratio of calculated dose to prescription dose at each calculation location.

	SSN	Head	Knee	Ankle	Overall
**Mean**	1.022	1.031	1.023	1.021	1.025
**Std Dev**	0.040	0.047	0.040	0.050	0.043

Since our treatment unit uses a Co‐60 source, we do not require a beam spoiler to achieve relatively uniform surface dose, as illustrated in Figure 3. Figure 3 also demonstrates the effects of this low energy on the dose profile along the beam axes. While the lack of penetration is visible in this profile, it does not represent a clinically relevant loss in dose uniformity with depth, even for thicker patients.

**FIGURE 6:**
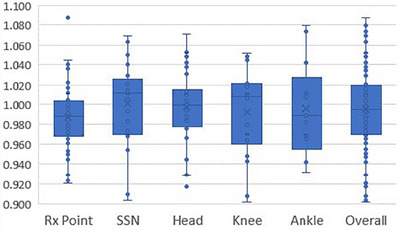
Results of in‐vivo dosimetry measurements by location. Boxes represent the interquartile range (IQR) and whiskers represent the maximum value within 1.5 times the IQR. The middle line within the box represents the median, and ‘X’ represents the mean value.

Results of in‐vivo diode measurements are presented in Figure 6 and Table 2. Figure 6 shows the distribution of measurements, including the interquartile range (IQR), median, and mean, along with outliers defined as measurements outside of 1.5 times the IQR. Table 2 provides mean values and standard deviations for the ratio of measured to calculated doses at each location. Measurements compare well with associated Monte Carlo point dose calculations, with a mean ratio of measured to calculated dose of 0.995 (standard deviation 0.040) for the 160 total data points. Mean values at each location are well within the standard deviation for all measurements with the exception of measurements on the head, for which the mean approaches the standard deviation. Including all in‐vivo dosimetry measurements, 124/160 (78%) agreed within ±5% of the calculated dose and 159/160 (99%) agreed within ±10%. Excluding measurements on the head, 83% of all measurements agree within ±5% and 99% agree within ±10%.

**TABLE 2:** Ratio of measured/calculated doses at each in‐vivo dosimetry location.

	Rx Point	SSN	Head	Knee	Ankle	Overall
**Mean**	0.987	1.001	1.037	0.993	0.996	0.995
**Std Dev**	0.033	0.046	0.038	0.045	0.048	0.040

A trend toward increased head diode reading with increasing patient length, increased SSN diode reading with increasing patient thickness, and increased MLD with increasing patient thickness were observed, however, none of these were statistically correlated based on regression analysis.

The lateral profile through the lung shown in Figure 4 represents absorbed dose along the horizontal dashed line through the axial slice shown in Figure 1(b). For reference, the edges of the lung contours along this profile are located at −9.8, −3.0, 3.5, and 9.5 cm, respectively. Lung block placement is verified with a movable flat panel imager located within the treatment couch with an estimated accuracy of 2 mm. However, lung block design likely has a more substantial effect on the MLD than the accuracy of placement. Luk et al demonstrate the substantial changes in the dose distribution and mean dose resulting from modifications in the design of lung blocks.^38^

## 4 DISCUSSION

Most TBI delivery techniques require patient‐specific compensation to achieve a uniform dose over the entirety of the patient. Our calculated mid‐depth doses for this cohort of patients with widely varying length and thickness show uniform dose at all chosen calculation points to within approximately 3% of the prescribed dose. However, there can be substantial variations from the prescribed dose in some locations. For example, the dose within the lateral aspects of the thorax and abdomen may substantially exceed the prescribed dose. This effect can be pronounced for patients with large fluctuations in thickness as a function of position, such as those with larger anterior‐posterior thickness. For the patient with the largest anterior‐posterior thickness in this cohort, approximately 5% of the total calculation volume received more than 10% greater than the prescribed dose. This region of increased dose is almost entirely within the lateral aspects of the patient's abdomen, thorax, and neck.

In‐vivo dosimetry results presented here compare well with previously published data,^9^, ^10^, ^11^, ^12^, ^13^, ^14^, ^39^, ^40^, ^41^ with the vast majority of results within 5% of the expected readings. We investigated potential causes for the increased diode readings on the head. Backscatter from the skull increases the measured dose on the surface of the head. This was evaluated with diode measurements on the surface of a phantom made of a stack of tissue equivalent plates. Measurements were first made on the surface of a 15 cm thick phantom made entirely of muscle tissue‐equivalent plastic. A subsequent measurement was then made on the surface of the same phantom but with a 1 cm plate of bone tissue equivalent plastic replacing the top 1 cm plate of muscle tissue equivalent plastic. Measurements made with the bone tissue equivalent plastic were approximately 2% higher than those without. However, we anticipate that this effect should be well modeled by the Monte Carlo treatment planning system and therefore should not result in a discrepancy between calculated and measured doses. The primary cause of the increased response shown here was quantified in a previous publication, which demonstrated a 5% increase due to scatter from the treatment couch head (the housing at the patient head end of the couch which contains the vertical drive mechanism) for the phantom end to end testing.^1^ This effect was further evaluated by repeated measurements at the same physical location in the beam but at varying distances from the couch head, and indicate a dose increase of 3%−4% resulting from scatter from the couch head for typical patient head locations. Accounting for this effect would change the overall ratio of measured to calculated dose in Table 2 from 1.037 to 1.002. This modest increase in dose is not considered clinically relevant and a clinical decision has been made not to move the location of the patient with respect to the treatment couch head as this would shift the patient's feet off the end of the treatment couch. As the purpose of the evaluation of in‐vivo measurements is to determine how accurately the measured doses agree with expected doses, the increase in dose resulting from the couch head is included in the data presented in Figure 6.

The MLD for this patient cohort can be compared to published estimates for the induction of radiation pneumonitis. For low dose rate single fraction delivery, Keane et al. suggest a threshold at approximately 8–9 Gy.^19^ For fractionated delivery at low dose rates, Lohr et al. suggest that lung doses of 9‐9.5 Gy can be administered safely in 12 fractions over 4 days, and Schneider et al. demonstrate a significant reduction in RP (25%–4.2% in pediatric patients) by decreasing the lung dose from 12 Gy to 11 Gy in 6 fractions over 3 days.^21^, ^23^ More recently, Shinde suggested a threshold of 8 Gy for fractionated high‐dose rate delivery using intensity‐modulated delivery of total marrow irradiation.^24^ For our low dose rate fractionated treatment regimen, a 9‐9.5 Gy threshold therefore seems the most appropriate and conservative. Our MLD results, ranging from 7.3‐9.3 Gy with a mean of 8.3 Gy, are therefore beneath this threshold. Numerous publications also choose a cutoff value for MLD and demonstrate a reduction in RP below this value. For example, Della Volpe and Pinnix use cutoff values of 9.4 and 13.5 Gy, respectively.^22^, ^28^ However, the dose per fraction for these two studies varies significantly and, using α/β = 3, these cutoff values represent EQD_2_ values of 11.5 Gy and 10.3 Gy, respectively. All of our patients have a MLD below these cutoff values both in terms of physical dose and EQD_2_. For pediatric patients, Children's Oncology Group protocols recommend shielding the lungs to receive < 8 Gy, a dose limit which is supported by prior studies of outcome associated with pediatric hematopoietic stem cell transplantation.^31^ Our cohort contains 4 pediatric patients. The MLD and associated EQD_2_ for these patients were 8.1 Gy and 7.0 Gy, respectively.

As a result of the low density of the lung, dose distributions without lung blocking result in significantly higher doses in the lungs than in the remainder of the body. Since the blocks do not cover the entire lung cross section in the beams eye view, peripheral regions of the lung can receive relatively large doses. This effect is observed in the high dose “tail” in the lung DVH shown in Figure 2. Given this dose distribution and the architecture of the lung, one may question whether the mean lung dose is the most relevant metric. Other DVH metrics, such as the volume receiving a particular dose, may ultimately be more useful in predicting RP. As examples, V_8Gy_, V_9Gy_, and V_10Gy_ for this patient were 44%, 28%, and 16%. However, the small number of patients for which we have accurate DVH data and the small number of observed cases of RP will make it difficult to determine the predictive capability of these metrics. Indeed, a recent analysis of patients treated at this facility from 1995 to 2017 indicates a grade ≥ 3 pneumonitis rate of <3% for patients receiving a fractionated low dose rate 12 Gy regimen.^42^ Furthermore, this cohort includes patients who received lung blocking (74%) and a resulting MLD of 8–9 Gy as well as those who did not receive lung blocking (26%) who received an MLD > 12 Gy. Unfortunately, these patients were treated in an era in which we did not have complete and/or accurate dose distribution data within the lungs, however, this illustrates the very small number of adverse events in the lungs and the associated difficulty in determining dose/volume metrics that are predictive of these events.

The heterogeneity in treatment planning and delivery techniques, the relative difficulty in accurately calculating the dose to the lung, and the relative severity of associated toxicities all highlight the importance of efforts to increase the accuracy and standardization of lung dose evaluation and reporting. Substantial variations have been reported in the dose delivered to the lung in comparison to the intended dose.^13^, ^18^, ^43^

Several authors have demonstrated a dose rate effect on RP rates.^44^, ^45^, ^46^ While a dose rate effect has not been definitively proven and several studies have observed no dose rate effect on RP, an overall evaluation suggests that, where present, it appears to be observed above approximately 15 cGy/min.^25^ The dose rate at the prescription location for patients in this study ranges between 7.5 and 15.5 cGy/min, with an estimated dose rate in the center of the lung of approximately 5−11 cGy/min and is therefore beneath this estimated threshold for dose rate effects. These values correspond to source activities of 12,200 Ci to 6,000 Ci. A thinner flattening filter is available and will replace the current flattening filter when treatment times become excessive. The thicker “attenuating” flattening filter was chosen for initial implementation of this unit to keep the dose rate below approximately 15 cGy/min.

## 5 CONCLUSIONS

In summary, we have presented the full dosimetric characteristics of TBI plans delivered using a dedicated Co‐60 TBI unit, demonstrating the ability to deliver a uniform dose to the entire patient without the need for a beam spoiler or patient‐specific compensation. Full dose distributions are calculated using an in‐house Monte Carlo treatment planning system and cumulative dose distributions are created for fields with and without lung blocking for two different patient orientations using the Eclipse treatment planning system. Cumulative dose distributions are then generated using deformable image registration to combine dose distributions from the two patient orientations. Sample distributions and profiles are provided to illustrate the plan characteristics and dose and DVH statistics are provided for a heterogeneous cohort of patients. Sample DVHs illustrate the characteristics of the dose distribution within the lungs, heart, and total body. A uniform dose of 12 Gy is prescribed to all non‐lung tissue within the patient, and nearly all of the patient volume outside of the lungs receives a dose within 10% of this prescription. Mean lung doses (MLDs) are below our estimated threshold for radiation pneumonitis for all patients in the cohort presented here. The mean MLD for this cohort was 8.3 Gy (range 7.3‐9.3 Gy), which when using an α/β value of 3, corresponds to an EQD2 of 7.3 Gy (range 6.2‐8.5 Gy). Calculated doses at five locations within the patient match the prescription dose to within 3.1% and are all within one standard deviation. In‐vivo dosimetric verification demonstrates excellent agreement with the calculated doses, with 78% of measurements within ±5% of the calculated dose and 99% within ±10%. These results characterize state‐of‐the‐art TBI planning and delivery processes using a dedicated TBI unit and hybrid in‐house and commercial planning techniques that provide comprehensive dosimetric data which has been accurately verified with in‐vivo dosimetry for this initial patient cohort.

## AUTHOR CONTRIBUTION

All authors meet the following criteria:

- Substantial contributions to the conception or design of the work; or the acquisition, analysis, or interpretation of data for the work; AND
- Drafting the work or revising it critically for important intellectual content; AND
- Final approval of the version to be published; AND
- Agreement to be accountable for all aspects of the work in ensuring that questions related to the accuracy or integrity of any part of the work are appropriately investigated and resolved.

## CONFLICTS OF INTEREST STATEMENT

The authors declare no conflicts of interest.
